# Chemical analysis of *Punica granatum* fruit peel and its in vitro and in vivo biological properties

**DOI:** 10.1186/s12906-016-1237-3

**Published:** 2016-07-30

**Authors:** Kaliyan Barathikannan, Babu Venkatadri, Ameer Khusro, Naif Abdullah Al-Dhabi, Paul Agastian, Mariadhas Valan Arasu, Han Sung Choi, Young Ock Kim

**Affiliations:** 1 Ethanopharmacology and Microbial Biotechnology Unit, Research Department of Plant Biology and Biotechnology, Loyola College, Nungambakkam Chennai, 600034 India; 2 Department of Botany and Microbiology, Addiriyah Chair for Environmental Studies, College of Science, King Saud University, Riyadh, 11451 Saudi Arabia; 3 Department of Emergency Medicine, College of Medicine, Kyung Hee University, Seoul, 02447 Republic of Korea; 4 Development of Ginseng and Medical Plants Research Institue, Rural Administration, Eumseong, 369-873 Republic of Korea

**Keywords:** *P. granatum*, α-Glucosidase inhibition, Antioxidants activity, Antimicrobial activity, *Caenorhabditis elegans*, GC-MS analysis

## Abstract

**Background:**

The medical application of pomegranate fruits and its peel is attracted human beings. The aim of the present study was to evaluate the in vitro α-Glucosidase inhibition, antimicrobial, antioxidant property and in vivo anti-hyperglycemic activity of *Punica granatum* (pomegranate) fruit peel extract using *Caenorhabditis elegans*.

**Methods:**

Various invitro antioxidant activity of fruit peel extracts was determined by standard protocol. Antibacterial and antifungal activities were determined using disc diffusion and microdilution method respectively. Anti-hyperglycemic activity of fruit peel was observed using fluorescence microscope for in vivo study.

**Results:**

The ethyl acetate extract of *P. granatum* fruit peel (PGPEa) showed α-Glucosidase inhibition upto 50 % at the concentration of IC50 285.21 ± 1.9 μg/ml compared to hexane and methanol extracts. The total phenolic content was highest (218.152 ± 1.73 mg of catechol equivalents/g) in ethyl acetate extract. PGPEa showed more scavenging activity on 2,2-diphenyl-picrylhydrazyl (DPPH) with IC50 value 302.43 ± 1.9 μg/ml and total antioxidant activity with IC50 294.35 ± 1.68 μg/ml. PGPEa also showed a significant effecton lipid peroxidation IC50 208.62 ± 1.68 μg/ml, as well as high reducing power. Among the solvents extracts tested, ethyl acetate extract of fruit peel showed broad spectrum of antimicrobial activity. Ethyl acetate extract supplemented *C.elegans* worms showed inhibition of lipid accumulation similar to acarbose indicating good hypoglycemic activity. The normal worms compared to test (ethyl acetate extract supplemented) showed the highest hypoglycaemic activity by increasing the lifespan of the worms. GC-MS analysis of PGPEa showed maximum amount of 5-hydroxymethylfurfural and 4-fluorobenzyl alcohol (48.59 %).

**Conclusion:**

In the present investigation we observed various biological properties of pomegranate fruit peel. The results clearly indicated that pomegranate peel extract could be used in preventing the incidence of long term complication of diabetics.

## Background

Diabetes mellitus is generally characterized by hyperglycemia that leads to disturbances in the metabolism of carbohydrates, lipids and proteins [1]. Chronic inflammation leads to obesity and it may be prevented by avoiding sugar containing food items. Nowadays insulin therapy is encouraged for the prevention of diabetes mellitus, but the therapy has several side effects like insulin resistance [2], anorexia nervosa, brain atrophy etc. Recently, the use of medicinal plants in modern medicine has been increased in order to prevent or to cure diseases [3–6]. At present food safety is an important concern due to the presence of food borne and other clinical pathogens. The quest for new antimicrobials have been taken into account by researchers worldwide due to the emergence of antibiotic resistant organisms and toxicity of synthetic drugs. Herbal plants and their extracts have been investigated in last few years due to the toxicological concerns of synthetic drugs [7, 8]. The secondary metabolites obtained from medicinal plants have also been investigated for their radical scavenging property.

*P. granatum* Linn. (Pomegranate) is abundantly present in India and belongs to family Punicaceae. Pomegranate peel contains tannins, flavonoids, polyphenols and some anthocyanins such as cyanidins and delphinidins [9]. Extracts from the peels of pomegranate has been proposed to play vital role in various pharmacological activities [10, 11]. The natural antioxidant food supplement will give the anti- aging process of skin, cells, tissues and organs. Antioxidants are present in certain fruits and vegetables that can protect human cells from oxidative damage and prevent aging of cells and body [12]. It reduces the incidence of tumors and infections. The plant constitutes gallotannic acid and the alkaloid such as isopelletierine, pelletierine, methypelletierine, psuedopelletierine, gallic acid, tannic acie, sugar, cacium oxalate, etc. However, the phytochemical constituents of the plant and antimicrobial activity of this plant have been reported in literature [13, 14]. It is very important to explore the findings of the research by investigation it in vivo and understanding its interactive effect.

*C. elegans* is a model organism that can be grown cheaply and in large numbers on plates. The worm is preferred over other model organisms especially mouse because they have a short life cycle of only 2 weeks which reduces the experimental cycles and the behaviour of individual cells can be studied because of its transparent body. Additionally, *C. elegans* genome have functional counterparts in humans which makes it a convenient model for human diseases especially diabetes study.

The nematode *Caenorhabditis elegans* contains abundant fat droplets in intestinal and hypodermal tissue. Compared to droplets in mammalian adipose tissue, which can expand to sizes of 100 μm [15], *C. elegans* lipid droplets are small, typically in the size range of 1–1.5 μm [16]. *C. elegans* has a multistep developmental process due to multicellularilty property. Regulation of lipid droplets inside the nematode using potential herbal extracts will yield key insights into the understanding of obesity, diabetes, and other metabolic diseases [17, 18]. From thios point of view, the present study was evaluated to determine antioxidant activity, α-Glucosidase activity, antimicrobial and antidiabetic property of pomegranate fruit peel extracts.

## Methods

### Collection of plant

The Healthy fresh pomegranate peel was collected Irula Tribe Women’s Welfare Society (ITWWS), Chengalpet, Tamil Nadu, India. The taxonomical identification of the plants was confirmed by Dr. Jeyajothi, botanist from Loyola College, Chennai, India. The plant was deposited under the vocher number LCH-74 in Loyola College, Chennai.

### Plant material and extraction

Peels of *P. granatum* (pomegranate) were shade dried and subsequently powdered. Five hundred grams of powdered peel was soaked in three different solvents (Hexane, ethyl acetate and methanol) at room temperature for 72 h in rotatory shaker (120 rpm). The powder and solvent were taken in the ratio of 1:3. The filtrates were further concentrated under reduced pressure at 40 °C and stored in a refrigerator at 2–8 °C for use in subsequent experiments.

### α-Glucosidase inhibition of solvent extracts

To analyse the α-Glucosidase inhibition, standard methodology was followed with some modifications [19, 20].

### Total phenolic content (TPC)

To analyse the total level of phenolic components, Folin–Ciocalteau method was followed with some modifications [20].

### DPPH radical scavenging assay of *P. granatum* fruit peel

DPPH quenching ability of *P. granatum* fruit peel hexane ether, ethyl acetate and methanol extracts was measured according to Hanato et al. [21].

### Evaluation total antioxidant activity of *P. granatum* fruit peel

The total antioxidant activity of *P. granatum* fruit peel was determined according to the method of [22].

### Lipid peroxidation assay

The evaluated the lipid peroxide properties of the extracts thiobarbituric acid method was followed [20].

### Reducing power activity of *P. granatum* fruit peel

The invitro reducing power activies of the different concnetrations of the plant extract were evaluated by following the standard methodology [3].

### Assessment of antimicrobial activity of *P. granatum* fruit peel

#### Test organisms

The following bacterial cultures were used to perform antibacterial test using MTCC and ATCC cultures: *Escherichia coli* MTCC 441*, Klebsiella pneumonia* ATCC 1705*, Streptomyces diastaticus* MTCC 1394 and *Enterococcus faecalis* MTCC 439.

Clinical isolates: *Enterobacter aerogenes, Klebsiella pneumoniae, Enterococcus faecalis, Staphylococcus epidermidis, Mycobacterium smegmatis* and *Escherichia coli* and other fungal strains.

### Disc diffusion method

The inhibition activity of the extracts against various pathogenic bacteria were determined by folling the standard methodology [3]. Plates were incubated overnight at 37 °C and then the zone of inhibition was measured in mm. All experiments were repeated in triplicate.

### Antifungal assays using broth micro dilution method

Antifungal activity was performed according to the standard reference method [23]. The antifungal agent, fluconazole was used as positive control and DMSO was used as negative control.

### *C. elegans* strains and culture conditions

The Bristol N2 (wild-type) *C. elegans* strain was used in this study and was obtained from Department of Genetic engineering, Madurai Kamaraj University, Tamil Nadu. It was maintained at 20 °C on nematode growth medium (NGM) agar plates. Plates were supplemented with *Escherichia coli* OP50 as nematode feed.

### Fluorescence microscope analysis for lipid accumulation in *C.elegans*

All worms used in this study were age-synchronized and the experimental animals were grown in liquid M9 medium and raised from eggs obtained by sodium hypochlorite treatment. The extracts of pomegranate fruit peel were made in three different concentrations (100 μg, 300 μg, and 500 μg/mL). They were added to dead OP50 (which were killed by autoclaving) in separate vials. They were then inoculated onto the NGM plates. Twenty worms were inoculated in each plate. Wild-type *C. elegans* were kept for 5 days under various glucose concentrations in the agar prepared as described above, harvested, and washed. Then drop of Nile Red (0.05 μg/mL) solution were added to the worms, which were then incubated for 30 min, washed with 25 % ethanol twice, and photographed in a Fluorescence microscope (Carl Zeiss Axioplan 2).

### Determination of triglycerides

Measurement of tryclycerides is used in screening of the lipid status of the worms. In vitro study was performed using ROBIniK Pritest Triglycerides assay kit using the treated *C. elegans* worms.

### Determination of life span

Synchronized worms were used for life span assay [24]. Twenty number of L4 worms were inoculated in5-fluorodeoxyuridine (FUDR40 mM) plate. The worms were grown at desired temperature (25 °C). The worms were scored every day to find out the activity of the plant extracts on them. The unmoved animals were considered as dead.

### GC –MS analysis

The individual compounds present in the extract was determined by GC-MS. The standard operating conditions were followed by the reported literature for GC-MS [3].

### Statistical analysis

All the results were analysed in Microsoft Excel 2007.

## Results and discussion

Medicinal plants and their extracts have exploited continuously by researchers in order to produce potential drugs of medicinal properties with reduced toxicity. In the line of this, we reported significant in vitro α-glucosidase inhibition activity of ethyl acetate extract of pomegranate peel. The α-glucosidase inhibiting potential of solvent extract such as hexane, ethyl acetate and methanol extracts from the fruit peel were tested and the results are summarized in Table 1. Ethyl acetate extract inhibited alpha glucosidase with the maximum value of 75.6 ± 2.03 % at 500 μg/ml with IC_50_ value of 285 ± 1.98 μg/ml. Alpha glucosidase inhibiting activity of fruit peel extracts are in the order of ethyl acetate > hexane > methanol.

**Table 1:** α-Glucosidase inhibition of extracts of *Punica granatum* (Pomegranate) fruit peal

Sample concentration (μg/ml)	*Punica granatum* fruit peel	
	α-Glucosidase inhibition	IC50 (μg/ml)
Hexane		
100	33.6 ± 1.9	349 ± 2.20
300	43.5 ± 2.0	
500	69.1 ± 2.3	
Ethyl Acetate		
100	38.7 ± 1.98	285 ± 1.98
300	51.5 ± 1.91	
500	75.6 ± 2.03	
Methanol		
100	20.7 ± 2.2	400 ± 2.58
300	38.1 ± 1.8	
500	62.0 ± 2.01	
Std (Acarbose)		
100	41.8 ± 1.60	205 ± 2.21
300	56.54 ± 1.91	
500	80.75 ± 2.08	

Each value represents the mean ± SEM of triplicate experiments

**Fig. 1:**
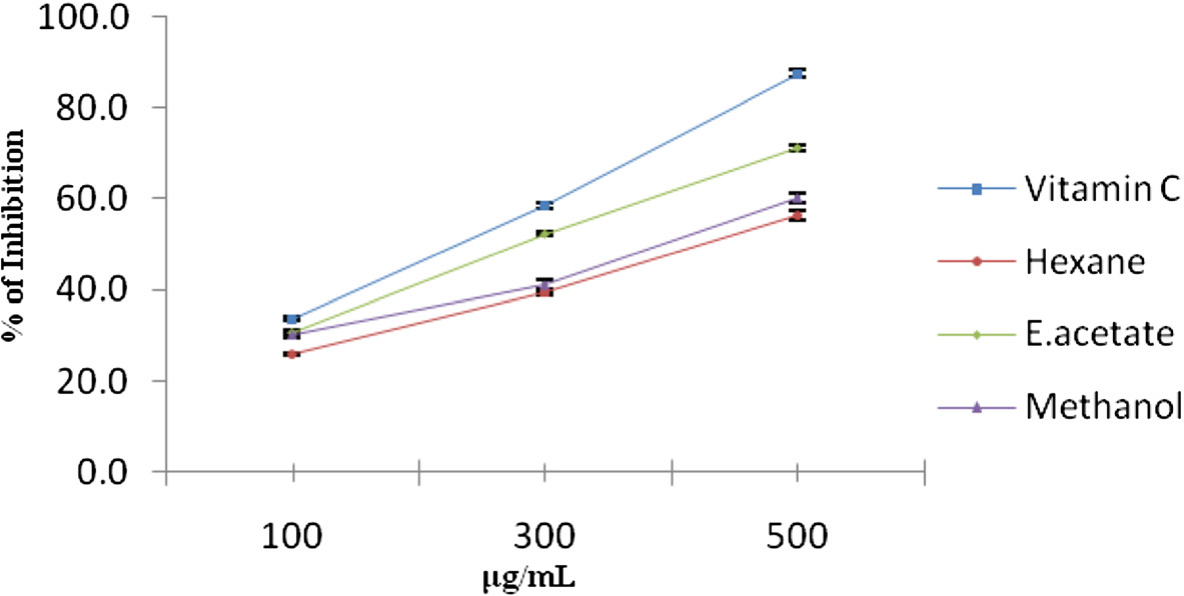
DPPH scavenging effect of hexane, ethyl acetate, and methanol extracts of *Punica granatum* (Pomegranate) fruit peel extracts and Vitamin C at different concentrations (100–500 μg/ml). Each value represents the mean ± standard deviation of triplicate experiments

Pomegranate fruit peel extract potently scavenged DPPH radicals similar to catechin, it is likely that peel extract possessed proton-donating ability and in association with a number of hydroxyl groups to stabilize free radicals [25, 26]. The results of this study suggest that the extracts contain phytochemical constituents that are capable of donating hydrogen to a free radical. Ethyl acetate extract of pomegranate peel has the ability to reduce the stable radical DPPH to diphenylpicryl hydrazine. The different concentrations of solvent extract of pomegranate peel showed antioxidant activities in a concentration-dependent manner (26–71.2 %) in the DPPH scavenging assay. Ethyl acetate extract (100–500 μg/ml) showed the highest activity (30.5–71.2 %). Figure 1 shows the scavenging effects of various extracts of pomegranate fruit peel on DPPH˙ in the following order: ethyl acetate > methanol > hexane.

**Fig. 2:**
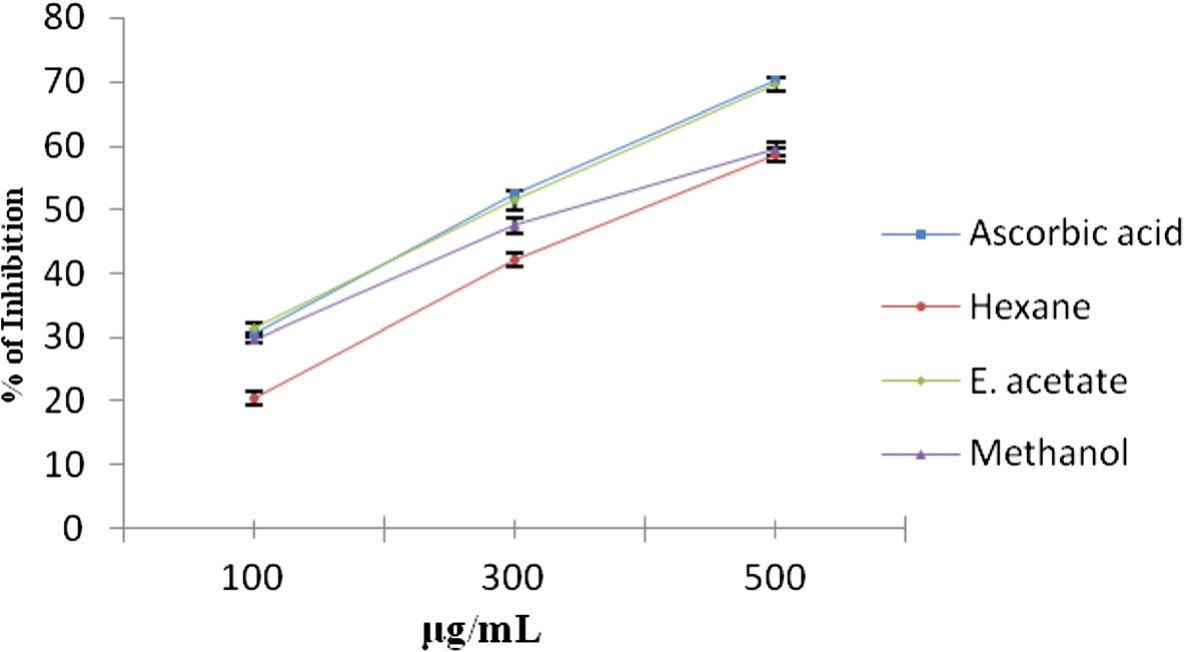
Total antioxidant activity of hexane, ethyl acetate, and methanol extracts of *Punica granatum* (Pomegranate) fruit peel extracts and Vitamin C at different concentrations (100–500 μg/ml). Each value represents the mean ± standard deviation of triplicate experiments

**Fig. 3:**
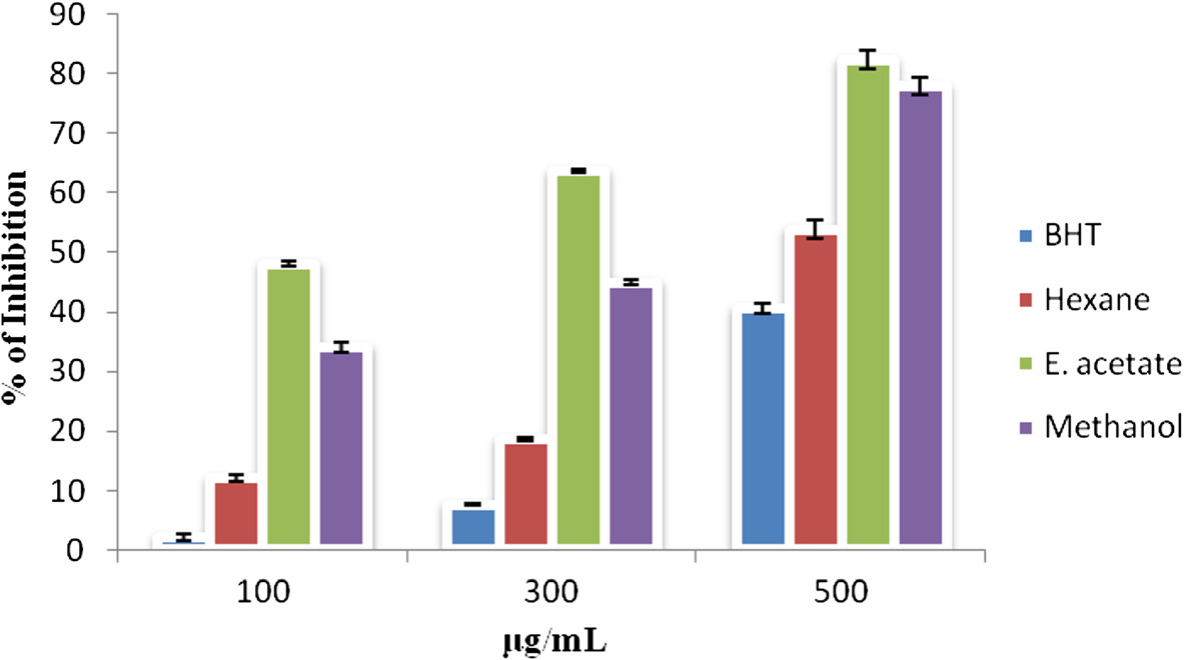
Reducing power of hexane, ethyl acetate, methanol extracts of *Punica granatum* (Pomegranate) fruit peel extractsand BHT at different concentrations (100–500 μg/ml). Each value represents the mean ± standard deviation of triplicate experiments

**Fig. 4:**
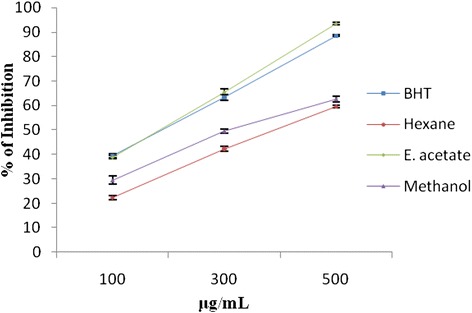
Lipid peroxidation scavenging effect of hexane, ethyl acetate, methanol extracts of *Punica granatum* (Pomegranate) fruit peel extracts and BHT at different concentrations (100–500 μg/ml). Each value represents the mean ± standard deviation of triplicate experiments

The total antioxidant capacity of various solvent extracts of pomegranate fruit peel at different concentrations (100–500 μg/ml) was found to be in the following order: ethyl acetate > methanol > hexane (Figs. 2, 3 and 4). Ethyl acetate extract (500 μg/ml) showed the highest total antioxidant activity (69.5 %).

The antibacterial activity of *P. granatum* fruit peel may be because of the presence of metabolic toxins or broad spectrum antimicrobial compounds that act against both Gram + ve and Gram –ve bacteria. The results of antibacterial activity of ethyl acetate extract of *P. granatum* (Pomegranate) fruit peel (PGPEa) against various human pathogens are listed in the Table 2. The MIC was significantly lower in ethyl acetate extracts that inhibits *T. rubrum* and *T. mentagrophytes* (31.25 μg/ml) (Table 3).

**Table 2:** Antibacterial activities of crude extracts of *Punica granatum (*Pomegranate*)* fruit peel

Name of the pathogen	Antibactrerial activity of extract (2.5 mg/ml)			Streptomycin (10 μg/disc)
	Zone of inhibition (mm)			
	Hexane	Ethyl Acetate	Methanol	
MTCC Isolates				
*Escherichia coli* MTCC 441	8 ± 1	16 ± 1	13 ± 2	24 ± 1
*Klebsiella pneumoniae* ATCC 1705	6 ± 1	9 ± 1	7 ± 1	11 ± 2
*Streptomyces diastaticus* MTCC 1394	13 ± 1	21 ± 1	17 ± 2	28 ± 1
*Enterococcus faecalis* MTCC 439	6 ± 1	12 ± 1	13 ± 1	14 ± 1
Clinical Isolates				
*Enterobacter aerogenes*	7 ± 1	19 ± 2	13 ± 1	22 ± 1
*Klebsiella pneumonia*	6 ± 1	14 ± 1	15 ± 1	19 ± 1
*Enterococcus faecalis*	7 ± 1	15 ± 1	16 ± 1	21 ± 1
*Staphylococcus epidermidis*	-	10 ± 1	-	16 ± 1
*Mycobacterium smegmatis*	10 ± 1	19 ± 2	16 ± 1	20 ± 1
*Escherichia coli*	-	10 ± 2	-	24 ± 1

The values are the average of three different experiments measuring the zone of inhibition (mm)

**Table 3:** Antifungal activities of crude extracts of *Punica granatum (*Pomegranate*)* fruit peel

S. No	Tested fungi	Hexane (μg/ml)	Ethyl acetate (μg/ml)	Methanol (μg/ml)	Fl (μg/ml)
1	*Curvularia lunata* 46/01	250	125	125	125
2	*T. rubrum* 57/01	250	31.2	125	250
3	*T. mentagrophytes* 66/01	250	31.2	125	250
4	*Botrytis cinerea*	250	250	125	250
5	*Aspergillus flavus*	250	31.2	125	62.5
6	*Aspergillus niger* MTCC 1344	250	62.5	125	250

The MIC values are the average of three different experiments measuring the μg/ml

*Fl* fluconazole, an antifungal agent

Various investigations were carried out to determine antioxidant, anticarcinogenic, and anti-inflammatory properties of pomegranate constituents [27–29]. Hajoori et al. [30] evaluated the antibacterial activity of different solvent extracts of *P. granatum* peel against human pathogens including four gram positive bacteria and six gram negative bacteria. According to Rathi et al. [31] *P. granatum* fruit peel can be used as an easily accessible source of natural antioxidant. They clearly demonstrated broad spectrum antimicrobial activity of pomegranate against bacteria. Additionally they mentioned that the presence of phytocompounds in the extracts including phenols, tannins and flavonoids as major active constituents may be responsible for these activities. Thus, the present study provides a strong direction for proper investigation of pomegranate fruit peel to explore molecules having antimicrobial properties against human pathogens. The presence of active inhibitors in pomegranate fruit peels including phenolics and flavonoids were revealed by phytochemical analysis as potent constituents.

The observed lifespan of *C. elegans*as model organism (control) was about 17 ± 1 days and the normal worms about 25 ± 1 days sillar to the other reported study [32]. When the observed result of the control was compared with that of the tests, we found that the ethyl acetate extract of *P. granatum* fruit peel showed the increased lifespan of the worms than that of the control i.e., about 24 ± 1 days. The second highest activity was shown for methanol extract of *P. granatum* (Table 4).

**Fig. 5:**
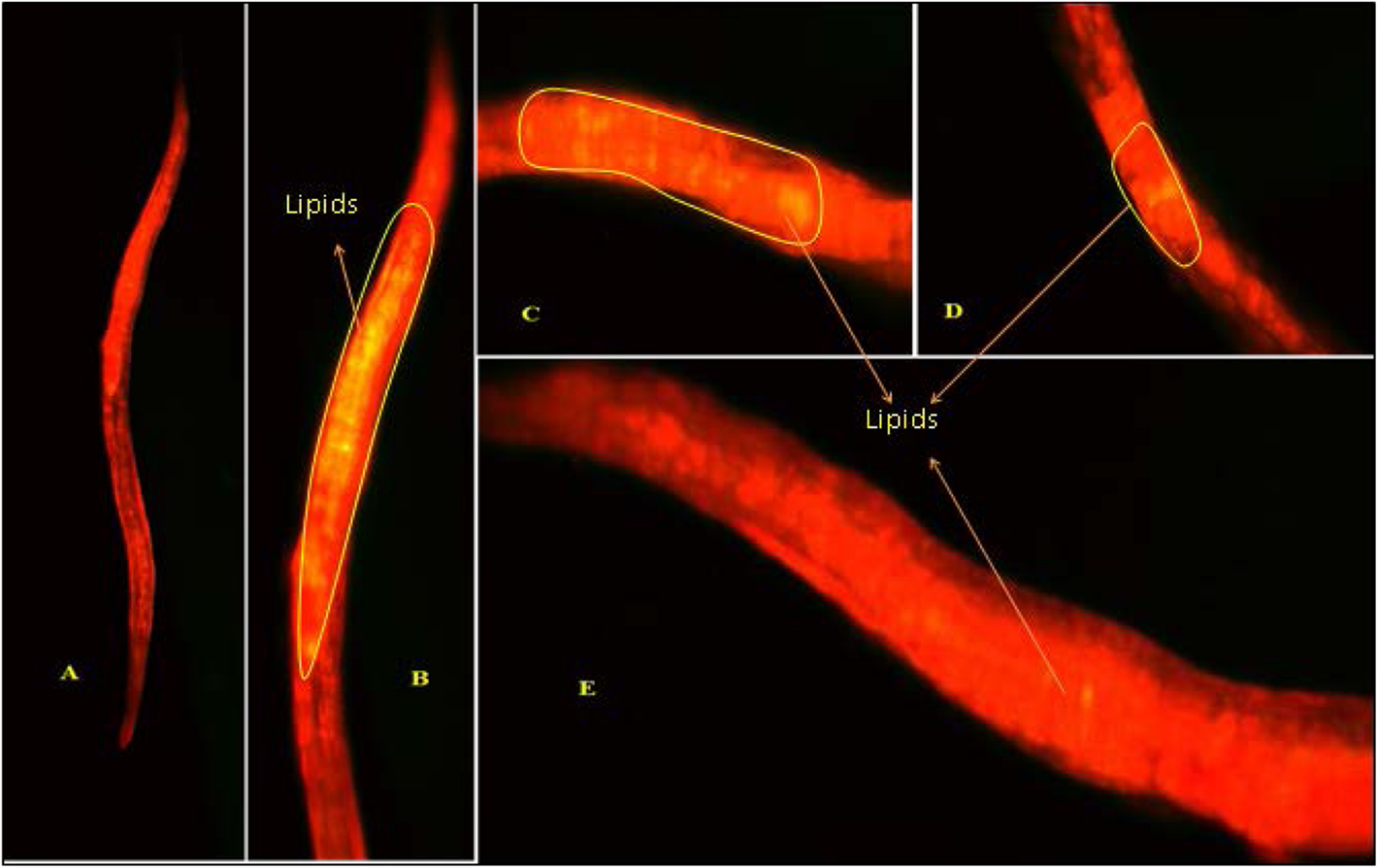
Lipid accumulation test in *C. elegans* under fluorescence microscope (Nile Red staining) – **a** Shows normal *C. elegans,* **b** Shows lipid accumulation in *C. elegans* in the presence of glucose solution, **c** Shows lipid accumulation in *C. elegans* in presence of PGPEa extracts extract (100 μg/ml), **d** Shows lipid accumulation in *C. elegans* in presence of PGPEa extracts extract (300 μg/ml), **e** Shows lipid accumulation in *C. elegans* in presence of PGPEa extracts extract (500 μg/ml)

**Fig. 6:**
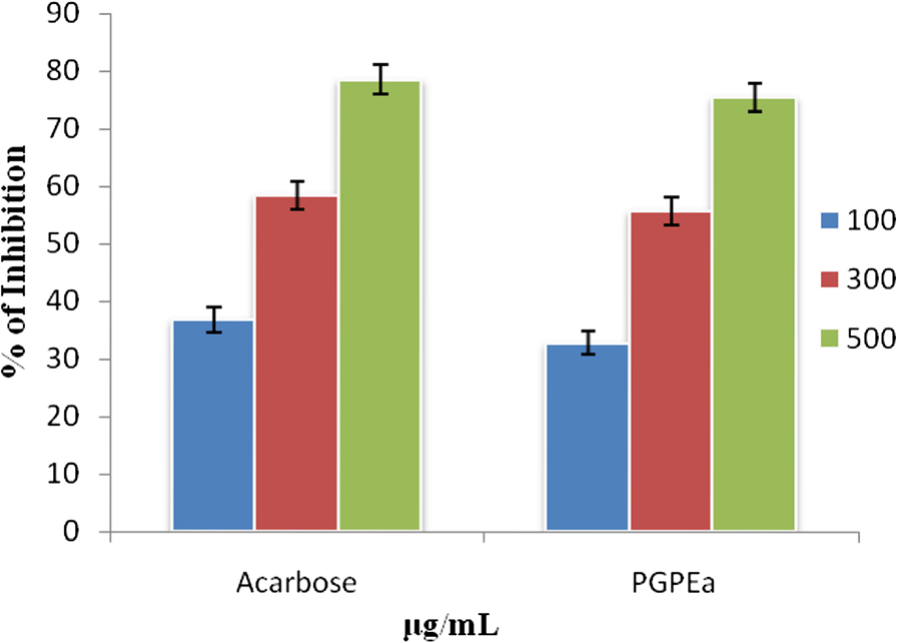
Lipid Quantification in *C. elegans* using Triglyceride assay for *Punica granatum* (Pomegranate) fruit peel extracts

**Fig 7:**
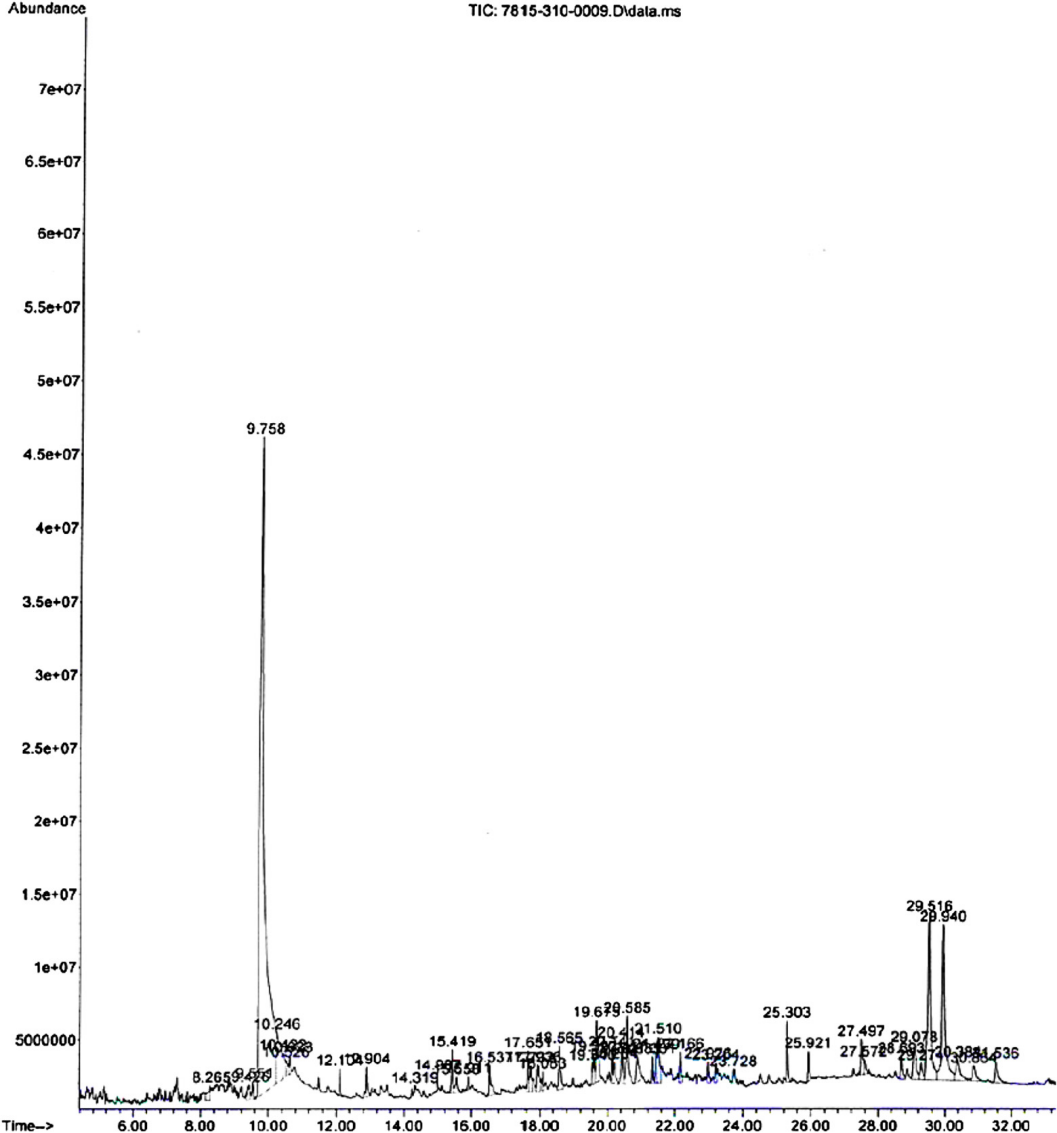
GC – MS Chromatogram of Ethyl acetate extracts of *Punica granatum* fruit peel extracts. 5-hydroxymethylfurfural and 4-fluorobenzyl alcohol metabolites were found to be most biologically active components (based upon the retention time) present in the crude extract

*C. elegans* provides a reliable tool to understand the changes in lipid accumulation in the body by glucose concentrations that are within the range observed in poorly controlled diabetic patients [33]. In the present study ethyl acetate extract of pomegranate fruit peel has the ability to reduce the lipid accumulation in the worm body. The fluorescence microscopic analysis clearly shows that ethyl acetate peel extract treated worms have accumulated less lipid compared to the normal worms (Figs. 5 and 6). Reduction in glucose level and lipid content in *C.elegans* indicates that ethyl acetate extract of pomegranate fruit peel has potential antidiabetic compounds which need to be isolated and identified through chromatographic techniques. Undoubtly ethyl acetate extracts of the test plant showed the presence of diverse molecules when subjected to GC-MS. In PGPEa a total of 48 compounds were detected, out ofwhich the maximum area was found for 5-hydroxymethylfurfural and 4-fluorobenzyl alcohol (Table 5) with a value of 48.59 % (Fig. 7).

**Table 4:** *C. elegans* Life span assay (Plate contain Glucose) of *Punica granatum* fruit peel

Extract	Concentration (μg/mL)	Lifespan (Days)
Hexane	100	18 ± 1
	300	18 ± 2
	500	19 ± 2
Ethyl Acetate	100	23 ± 1
	300	24 ± 2
	500	25 ± 2
Methanol	100	20 ± 1
	300	21 ± 1
	500	22 ± 1
Control	100	17 ± 1
	300	18 ± 2
	500	18 ± 1

Each value represents the mean ± SEM of triplicate experiments

**Table 5:** Phytocomponents identified in the ethyl acetate extracts of *Punica granatum* fruit peel (PGPEa) (GC-MS Study)

S. No	Chemical name	Retention time	% of Area
1	2-furan Carboxamide, N-(3-nitrophenyl)- 1-propanone, 1-(2-furanyl)-4-Pyridinol	8.266	0.60
2	4H-Pyran-4-one, 3,5-dihydroxy-2-methyl, 4H-Pyran-4-one, 3,5-hydroxy-2-methyl.	9.425	0.62
3	Benzne, 1,3-bis(1,1-dimethylethyl, Benzne, 1,4-bis(1,1-dimethylethyl	9.552	0.31
4	5-hydroxymethylfurfural, 4-fluorobenzyl alcohol	9.760	48.59
5	5-hydroxymethylfurfural, 4-mercaptophenol	10.242	2.99
6	5-hydroxymethylfurfural, 4- fluorobenzyl alcohol	10.525	0.30
7	5-hydroxymethylfurfural, 4-fluorobenzyl alcohol	10.621	0.39
8	Bicycol [7.2.0] undec-4-ene, 4,11,11-trimethyl-8-methylene, [1R-(1R,4z,9S)]- caryphyllene	12.107	0.38
9	Hexadecane, 1-iodo- Hexadecane Nonane	12.902	0.59
10	Z-8- Hexadecane, 9-Eicosene, (E)- n-Pentadecanol	14.321	0.14
11	Copaene, alpha. Cubebene	14.989	0.29
12	Hexadecane, 2-Bromotetradecane	15.420	0.90
13	Heneicosane, 11-pentyl-Docosane, 11-butyl-Tridecane	15.561	0.54
14	Nonadecane, 9-methyl-Nonane, 5-butyl-Heptadecane	15.911	0.27
15	Z-8-Hexadecane, Pentafluropropinonic acid, 4-hexad ecyl ester	16.535	0.94
16	Heneicosane, Eicosane	17.649	0.63
17	Nonadecane, 9-methyl, 7,9-Di-tert-butyl-1-oxaspiro(4,5)deca-6,9-diene-2,8-dione.	17.731	0.68
18	Pentadecanoic acid, 14-methyl easter, Hexadecanoic acid, methyl easter	17.939	0.81
19	Nonadecane, 9-methyl, Eicosane, Pentacosane	18.080	0.34
20	1-heneicosyl formate, Cyclooctacosane, 9-Tricosen, (Z)-		
21	Tetracosane, Octodecane, Hexadecane	19.543	0.48
22	Triacontane, 1-bromo-1-Chloroeicosane Heptadecane	19.595	0.59
23	Dodecane, 2,6,11-trimethyl-docosane, 7-hexyl-Tetracosane	19.677	1.98
24	Linoleic acid ethyl ester n-Propyl 9, 12-octadecadienoate 9, 12-octadecadienoic acid, ethyl ester	20.137	0.53
25	1-nonadecene, 9-Trocosene, (Z)- Bacchotricuneatin	20.204	0.63
26	1-nonadecene, 9-Trocosene, Z-5- Nonadecene	20.412	0.71
27	Tetracosane, Octadecane, Heptadecane	20.457	0.50
28	6-octen-1-ol, 3,7-dimethyl acetate Phytol, acetate 1,2-15, 16-Diepoxyhexadecane	20.583	2.02
29	3,5,7-Tricyclopropyl-5,6-dihydro-5-methyl-1,2(4H)-diazepineOctanoic acid, but-3-yn-2-yl ester Ethisterone	20.888	1.14
30	Triacontane, Heptadecane, Octacosane	21.333	0.39
31	3H-Cyclodeca[b]furan-2-one, 4, 9-dihydroxy-6-methyl-3, 10-dimethylene-3a, 4, 7, 8, 9, 10, 11, 11a-octahydro-Bicyclo[10.1.0]trideca-4, 8-diene-1 3-carboxamide, N-(3-chlorophenyl)-1H-2, 8a-Methanocyclopenta[a]cyclopropa[e]cyclodecen-11-one, 1a, 2, 5, 5a, 6, 9, 10, 10a-octahydro-5, 5a, 6-trihydroxyl-1, 4-bis(hydroxymethyl)-1, 7, 9-trimethyl, [1S-(1.alpha., 1a.alpha., 2.alpha., 5.beta., 5a.beta., 6.beta., 8a.aipha., 9.alpha., 10a.alpha.)]	21.437	0.83
32	Heptadecane, 3-methyl- OctadecaneNonadecane	21.512	1.41
33	Octacosane, Tetracosane	22.165	0.81
34	Eicosane, Triacotane, Octadecane	22.975	0.42
35	Hexatriacontane, Octadecane, 1-iodo-Tetratetracontane	23.205	0.37
36	1-hexacosene, 9-hexacosene, E-15-heptadecenal	23.725	0.37
37	Squalene	25.300	1.07
38	Eicosane, Heneicosane,	25.924	0.56
39	Vitamin E (+)-gamma- Tocopherol, O-methyl-dl-alpha.- Tocopherol	27.499	0.96
40	CyclobarbitalTris(tert-butyldimethylsilyloxy)arsane, 1H-Indole-2-carboxylic acid, 6-(4- ethoxyphenyl)-3-methyl-4-oxo-4, 5, 6, 7-tetrahydro isopropyl ester	27.573	0.41
41	2, 4-Cyclohexadien- 1-one, 3, 5-bis, 1-dimethylethyl)- 4-hydroxy- Tetrasiloxane, decamethyl- Benz[b]-1, 4-oxazepine-4(5H)-thione, 2, 3-dihydro-2, 8-dimethyl	28.695	0.69
42	Anthracene, 9, 10- dihydro-9, 9, 10-trimethyl-1H- Indole, 1-methyl-2-phenyl-Ethanone, 2-(2-benzothiazolylthio)-1-(3, 5-dimethylpyralyl)	29.082	1.52
43	N-Methyl-1-adamantaneacetamide Arsenous acid, tris(trimethylsilyl) ester, Benzo[h]quinolone, 2, 4-dimethyl	29.275	0.69
44	9, 19-cyclolanost-24-en-3-ol, Lanosterol, Lanost-7-en-3-one	29.512	7.37
45	Tirucallol, Lanosterol, D:B-Friedo-18, 19-secolup-19-ene, 10-epoxy	29.943	7.58
46	1, 2-Bis(trimethylsilyl) benzene, 4-Dehydroxy-N-(4, 5-methylenedioxy- 2-nitrobenzylidene) tyramineBenzo[h]quinolone, 2, 4-dimethyl	30.382	1.48
47	1H-Indole, 1-methyl-2-phenyl- Arsenous acid, tris(trimethylsilyl) ester, Cyclotrisiloxane, hexamethyl	30.864	0.86
48	5-methyl-2-phenylindozine (1H) Pyrrole-3-carboxylic acid, 5-[cyano(4-morpholinyl) methyl]-1-(methoxymethyl), methyl ester 2- (Acetoxymethyl)-3-(methoxycarbonyl) biphenylene	31.533	1.06

## Conclusion

It is concluded that the ethyl acetate extract of pomegranate fruit peel contained considerable levels of phenols and flavonoids which are responsible for α-glucosidase inhibition and antioxidant activities. These in vitro assays also indicate that the PGPEa not only has potential antidiabetic and natural antioxidant compounds but also has the ability to increases the life span of *C. elegans.* Future studies are required to prove whether the process described in *C. elegans* can be translated to the situation in diabetic patients. 5-hydroxymethylfurfural and 4-fluorobenzyl alcohol compounds detetcted in GCMS might plaied the role in antioxidant and antimicrobial potentials of the extracts.

## Abbreviations

GC-MS, gas chromatography–mass spectrometry
